# Stratification of pseudoprogression and true progression of glioblastoma multiform based on longitudinal diffusion tensor imaging without segmentation

**DOI:** 10.1118/1.4963812

**Published:** 2016-10-05

**Authors:** Xiaohua Qian, Hua Tan, Jian Zhang, Weilin Zhao, Michael D. Chan, Xiaobo Zhou

**Affiliations:** Department of Radiology, Wake Forest School of Medicine, Medical Center Boulevard, Winston-Salem, North Carolina 27157; Department of Radiation Oncology, Wake Forest School of Medicine, Medical Center Boulevard, Winston-Salem, North Carolina 27157; Department of Radiology, Wake Forest School of Medicine, Medical Center Boulevard, Winston-Salem, North Carolina 27157

**Keywords:** glioblastoma multiform, pseudoprogression, longitudinal DTI, spatio-temporal dictionary learning, discriminative sparse matrix

## Abstract

**Purpose:**

Pseudoprogression (PsP) can mimic true tumor progression (TTP) on magnetic resonance imaging in patients with glioblastoma multiform (GBM). The phenotypical similarity between PsP and TTP makes it a challenging task for physicians to distinguish these entities. So far, no approved biomarkers or computer-aided diagnosis systems have been used clinically for this purpose.

**Methods:**

To address this challenge, the authors developed an objective classification system for PsP and TTP based on longitudinal diffusion tensor imaging. A novel spatio-temporal discriminative dictionary learning scheme was proposed to differentiate PsP and TTP, thereby avoiding segmentation of the region of interest. The authors constructed a novel discriminative sparse matrix with the classification-oriented dictionary learning approach by excluding the shared features of two categories, so that the pooled features captured the subtle difference between PsP and TTP. The most discriminating features were then identified from the pooled features by their feature scoring system. Finally, the authors stratified patients with GBM into PsP and TTP by a support vector machine approach. Tenfold cross-validation (CV) and the area under the receiver operating characteristic (AUC) were used to assess the robustness of the developed system.

**Results:**

The average accuracy and AUC values after ten rounds of tenfold CV were 0.867 and 0.92, respectively. The authors also assessed the effects of different methods and factors (such as data types, pooling techniques, and dimensionality reduction approaches) on the performance of their classification system which obtained the best performance.

**Conclusions:**

The proposed objective classification system without segmentation achieved a desirable and reliable performance in differentiating PsP from TTP. Thus, the developed approach is expected to advance the clinical research and diagnosis of PsP and TTP.

## 1. INTRODUCTION

Glioblastoma multiforme (GBM) is the most common and aggressive primary brain tumor, with an incidence of approximately 3 cases per 100000 people life-years in Europe and North America.^1^ The median survival for GBM patients is about 14–16 months and the average 2-yr survival rate is only 26%–33%, even though they have received standard care including surgical resection followed by concurrent radiotherapy and chemotherapy with temozolomide.^1^ The radiotherapy and chemotherapy are effective for the treatment of GBM, but also increase the difficulty of distinguishing true tumor progression (TTP) and pseudoprogression (PsP).^2^ PsP is a subacute and post-treatment reaction with increased contrast enhancement and vasogenic edema that mimics tumor progression at the tumor site or resection margins, but subsequently regresses or remains stable.^3,4^ The incidence of PsP is around 20% in all GBM patients with standard treatment.^2^

Clinically, it is crucial to differentiate PsP from TTP because management strategies are different. The conventional magnetic resonance imaging (MRI) is unable to distinguish PsP from TTP since these two entities have similar intensity and shapes on MRI. Currently, the only method for distinguishing between PsP and TTP is to perform follow-up MRI examinations based on the changes in the lesion site. It usually takes several months to obtain an accurate diagnosis, which will result in a critical delay for the clinical management of patients. Although the pathological confirmation of PsP may be helpful, brain tumor biopsies are not typically done in clinical practice, since the procedure is invasive, carries increased risk for the given chemoradiotherapy, and may lead to delays in the treatment due to the process of wound healing. Therefore, there is an urgent need to develop novel methods for the early diagnosis of PsP and TTP.

Over the last decade, distinguishing between PsP and TTP has been recognized as a significant issue, and many efforts have been devoted to explore genetic biomarkers and imaging features to address this need. Several genetic and molecular markers involved in GBM have been associated with the development of PsP, including MGMT promoter methylation,^5^ Ki67 expression,^6^ IDH1 mutation,^7^ and p53 mutation.^8^ However, their predictive values remain debatable, and the clinical significance of these associations needs to be confirmed.^2,9–11^

Functional MRIs, including diffusion-weighted imaging (DWI), perfusion-weighted imaging (PWI), and diffusion tensor imaging (DTI), have been extensively investigated for their ability to discriminate between PsP and TTP based on quantitative parameters, such as relative cerebral blood volume (rCBV),^12–14^ apparent diffusion coefficient (ADC),^13–15^ fractional anisotropy (FA),^12^ and the ratio of the area under the time signal-intensity curves (AUCR).^16^ Both volumetric plasma volume (Vp) and time-dependent leakage constant (Ktrans) volumetric histogram metrics have been developed using the dynamic enhancement MRI and a parametric response map (PRM) of dynamic susceptibility contrast PWI.^17,18^ Chen *et al.* generated 5 Gy-level co-occurrence matrix texture maps of contrast, energy, entropy, correlation, and homogeneity to differentiate PsP and TTP on MRI scans.^19^ These studies directly extracted the imaging data from the selected region of interest (ROI) and selected one parameter or a combination for differentiation. However, these schemes had limited success, for several reasons. First, the ROI of the lesion was manually or semiautomatically segmented according to operator-dependent judgment, which is both subjective and labor-intensive. Second, the analysis of basic image features, such as mean, median metrics, and histogram, cannot capture subtle differences between PsP and TTP in MRIs. In addition, those studies were focused on evaluating various MRIs using different parameters, not developing an objective and automated classification system for PsP and TTP.

Thus, we developed a novel objective classification system using dictionary learning based on longitudinal DTI for effectively differentiating PsP and TTP. DTI measures the directionality of proton movement, which can be used to compute the maps of FA. Since brain tissues with treatment effects have lower FA values compared with recurrent tumors,^20^ we posited that DTI would have the potential to be used for differentiating between PsP and TTP.^21^ Because follow-up scanning is the optimal method for the diagnosis of PsP and TTP, we employed longitudinal DTI methods in our classification system. In most conventional classification systems, the accurate segmentation of ROIs is needed to determine the performance of classification. It is a challenge to automatically segment ROIs to distinguish between tumor recurrence and treatment-induced effects of GBM in DTI, although some GBM segmentation and registration tools have been well established.^22,23^ To address this issue, in this study, we directly extracted features from the sparse matrix using a dictionary learning scheme. Therefore, our classification system did not suffer from the dilemma of segmentation.

Dictionary learning and sparse coding is a branch of signal processing and machine learning aimed at discovering a frame in which some of the training data admit a sparse representation.^24,25^ Dictionary learning-based methods have been widely used in different applications, such as image denoising,^26^ image restoration,^27^ and classification.^28^ K-SVD is one of the most comprehensive dictionary learning methods, but it is not specifically designed for classification tasks.^29^ Recently, other dictionary learning methods incorporating the label information in the training set were developed to produce a classification-oriented dictionary.^30–33^ These methods have yielded good classification performances in face recognition and natural scene classification.

Inspired by these state-of-the-art dictionary learning algorithms, we applied a classification-oriented and spatio-temporal dictionary learning scheme in our classification system. First, we analyzed the longitudinal DTI directly by using the spatio-temporal volume (2D + *t*) to extend 2D spatial patches without relying on an intermediary descriptor. The sparse dictionaries were defined by the 3D spatio-temporal representation of the FA. Second, PsP and TTP display subtle differences in DTI, and their commonly shared features do not contribute to their discrimination. Therefore, we explicitly learned the PsP/TTP-specific dictionaries and the common pattern dictionary and encoded the DTIs using these dictionaries. We then combined the corresponding sparse coefficients of the PsP-specific and TTP-specific dictionaries to construct a new sparse matrix with a strong discriminating power. After that, we extracted features from the sparse matrix using the max.pooling technique, and evaluated and selected the most discriminating features by our feature scoring system.^34,35^ Finally, we classified 35 patients with GBM into PsP and TTP categories using the established support vector machine tool LIBSVM (Ref. 36) and validated this classification system using tenfold cross-validation (CV) experiments and area under receiver operating characteristic (AUC) analysis.

Overall, the major contributions of our study can be summarized as follows. First, we are the first group to develop an objective classification system for the differentiation of PsP and TTP based on the longitudinal DTI, which can improve the accuracy and efficiency of diagnosis and avoid operator dependence and inter-observer variation in clinics. Second, the dictionary learning strategy makes the classification system circumvent the segmentation of ROIs. Finally, we constructed a novel sparse matrix to exclude the common pattern shared by PsP and TTP. Thus, this sparse matrix possesses a strong discrimination power for classification.

## 2. MATERIALS AND METHODS

**FIG. 1.**
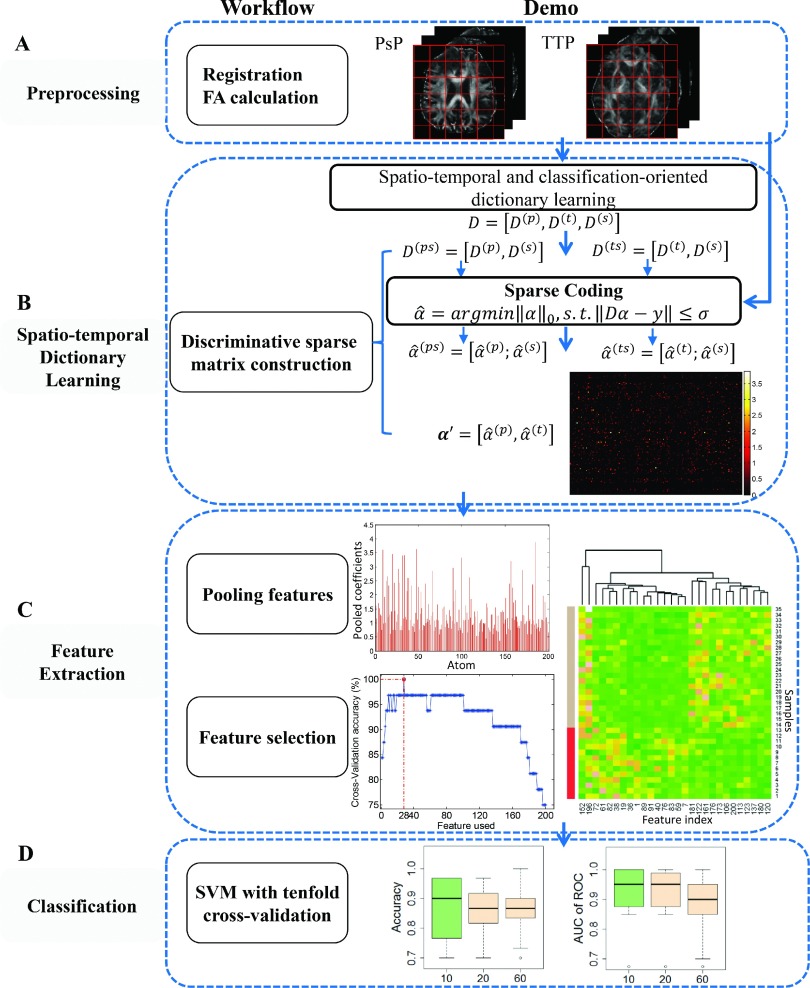
Schematic for the classification of PsP and TTP in patients with GBM based on spatio-temporal dictionary learning without segmentation. *D* = dictionary; *α* = sparse coefficient. PsP, TTP, and the shared patterns are denoted by superscript *p*, *t*, and *s*, respectively.

Since DTI-FA values in the lesion area of PsP are lower than that in the TTP region, we developed a novel spatial–temporal discriminative dictionary learning method to capture the difference of FA between PsP and TTP, thereby distinguishing PsP from TTP. As shown in Fig. 1, the study had four phases: preprocessing, spatio-temporal dictionary learning, feature extraction, and classification. In the spatio-temporal dictionary learning phase, a discriminative sparse matrix was constructed by excluding the shared pattern of categories. Then, the local features were selected and evaluated by a feature scoring system from the pooled features based on sparse coefficients in the feature extraction phase. Finally, PsP and TTP were classified using standard support vector machine (SVM) techniques with tenfold CV.

### 2.A. Data collection

This study was approved by the Institutional Review Board of Wake Forest School of Medicine. The clinical records and longitudinal DTI from 35 GBM patients (13 with PsP and 22 with TTP) were collected at the Wake Forest School of Medicine. All of these patients received standard care, including surgical resection followed by concurrent radiotherapy and chemotherapy with temozolomide. The enrolled patients received a similar dose (around 60 Gy) of conformal radiotherapy. Along with radiotherapy and chemotherapy, the patients underwent DTI scans (scanner: SIMCGEMR, GE Medical systems) each two or three months for postsurgical monitoring. The diagnosis of PsP and TTP was based on the follow-up imaging data and clinical experience of the physicians in charge. Biopsies were also performed in seven patients for further confirmation. For each patient, we retrospectively selected successive DTI scans at three time-points. The third time point, i.e., the latest one, was the time when a patient was diagnosed with PsP or TTP. The corresponding clinical record included age, sex, date of surgery, date of failure or progression, pseudoprogression, and date of death.

### 2.B. Preprocessing DTI data

We computed the FA value for each voxel in DTI using FSL software.^37^ Prior to this computation, current-induced distortions and subject movements were corrected, and the skull and skin of the head were removed. Then, we linearly registered the reconstructed FA brain to the standard brain template FMRIB58 in FSL. Each section of registered volumetric FA data owned the identical resolution and number of slices; thus, the same voxels across longitudinal images were properly registered to one another.

### 2.C. Construction of spatio-temporal patches for dictionary learning

**FIG. 2.**
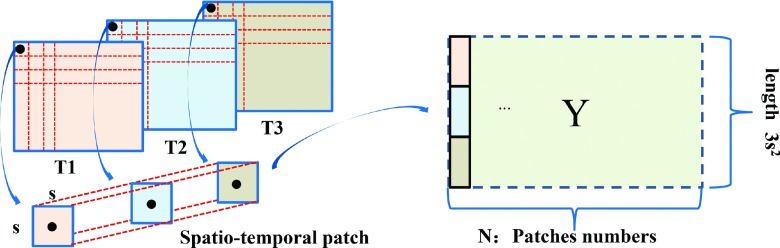
Construction of spatio-temporal patches for dictionary learning. *T* = time point.

The longitudinal DTI for GBM patients provides the dynamic changes of pathology based on FA value, which can be used for the diagnosis of PsP and TTP. Since the amount of longitudinal data is huge, learning a dictionary directly over the entire set of data is difficult. Thus, we adopted a 3D patch-based dictionary learning approach from spatio-temporal volumes. We define a spatio-temporal image patch as a succession of 2D image patches. First, an axial image in a volume with the largest cross-sectional lesion area was selected from each longitudinal DTI. Let *y* be a 3D patch of size *s* × s*t, where *s* and *t* are the patch sizes in spatial and temporal dimensions, respectively. This patch *y* was then converted to the column-vector of length *m* = *s*^2^*t*, as shown in Fig. 2. *y* = (*a_i_*)_*i*∈[1,*m*]_ with *a_i_* gray level (i.e., FA value) associated to the pixel *i*. Each patch is normalized as $y_{\text{norm}}=\left(y-\bar{y}\right)/{∥y∥}^{2}$, where $\bar{y}$ is the mean of the vector *y* and ${∥y∥}^{2}$ is the *L*_2_-norm. To capture spatio-temporal structure in the proposed scheme, we adjusted *s* to elastically define the contributions of spatial dimension.

### 2.D. Specific dictionary learning for PsP and TTP

The difference between PsP and TTP in DTI is subtle. PsP has slightly lower FA values than TTP in the pathological area, and there is considerable similarity between PsP and TTP in normal regions. To capture the fine distinctions, we learned specific dictionaries for PsP and TTP, respectively, to describe the class-specific characteristics. Simultaneously, we learned a shared dictionary to describe the common features of PsP and TTP.

Mathematically, a matrix is composed of spatio-temporal patches $Y=\left[y_{1},y_{2},\ldots ,y_{N}\right]\in R^{m\times N}$, where *N* being the size of the training set. Dictionary learning aims to seek a dictionary and the corresponding sparse coefficient so that each patch *y_i_* can be sparsely represented by the atoms in this dictionary. The learning process can be described by the following optimization problem:

$$\underset{D,A}{\min}\overset{N}{\underset{i=1}{\sum }}\left\{\frac{1}{2}{∥y_{i}-D\alpha_{i}∥}_{2}^{2}+\lambda_{1}{∥\alpha_{i}∥}_{1}\right\}.$$ (1)

The parameter *λ*_1_ balances the trade-off between data fidelity and sparsity regularization. The dictionary $D\in R^{m\times K}$ and corresponding sparse coefficients $A\in R^{K\times N}$ are both learned. Each column of *D* and *A* is denoted as $d_{i}\left(i=1,\ldots ,K\right)$ and $\alpha_{j}\left(i=1,\ldots ,N\right)$, respectively. The dictionary *D* and coefficients *A* can be used effectively to represent the data; however, they are not developed for classification tasks.

In this study, we had two categories, i.e., PsP and TTP, and $Y=\left[Y^{\left(p\right)},Y^{\left(t\right)}\right]\in R^{m\times N}$, where *N* = *N_p_* + *N_t_*. We denoted the PsP and TTP class as superscript or subscript *p* and *t*, respectively. $Y^{\left(p\right)}\in R^{m\times N_{p}}$ represents the data from the PsP class. As we observed in the DTI, the PsP and TTP classes had lots of shared features, which do not contribute to the discrimination. To seek the essential differences between these two categories, the class-specific characterizations and common patterns must be separated. Therefore, we directly learned PsP- and TTP-specific dictionaries *D*^(*p*)^ and *D*^(*t*)^ to obtain the classification-oriented characterizations, and learned the shared dictionary *D*^(*s*)^ to separate the common patterns. We denoted the overall dictionary as $D=\left[D^{\left(p\right)},D^{\left(t\right)},D^{\left(s\right)}\right]\in R^{m\times K}$, in which *K* = *K_p_* + *K_t_* + *K_s_*.

To obtain the specific dictionary *D*, we derived the following objective function:

argminD,A∑i=1NpOpi+∑j=1NtOtj+η∑i,ji≠j3DiTDjF2, (2)

where

$$O_{p}\left(i\right)={∥y_{i}-D\alpha_{i}∥}_{2}^{2}+\lambda_{1}{∥\alpha_{i}∥}_{1}+{∥y_{i}-D^{\left(p\right)}\alpha_{i}^{\left(p\right)}-D^{\left(s\right)}\alpha_{i}^{\left(s\right)}∥}_{2}^{2}+{∥\alpha^{\left(t\right)}∥}_{2}^{2},$$ (3)

$$O_{t}\left(j\right)={∥y_{j}-D\alpha_{j}∥}_{2}^{2}+\lambda_{1}{∥\alpha_{j}∥}_{1}+{∥y_{j}-D^{\left(t\right)}\alpha_{j}^{\left(t\right)}-D^{\left(s\right)}\alpha_{j}^{\left(s\right)}∥}_{2}^{2}+{∥\alpha^{\left(p\right)}∥}_{2}^{2},$$ (4)

where $\alpha =\left[\alpha^{\left(p\right)};\alpha^{\left(t\right)};\alpha^{\left(s\right)}\right]\in R^{K\times N}$. $\alpha^{\left(p\right)}\in R^{K_{p}}$ is the coefficient corresponding to *D*^(*p*)^. ${∥y_{i}-D^{\left(p\right)}\alpha_{i}^{\left(p\right)}-D^{\left(s\right)}\alpha_{i}^{\left(s\right)}∥}_{2}^{2}$, i.e., the third term of Eq. (3), requires the class-specific dictionary *D*^(*p*)^, in conjunction with the shared dictionary *D*^(*s*)^, to well represent PsP data $\left(Y^{\left(p\right)}\right)$. We also forced the coefficients to be zero, except parts corresponding to the specific and shared dictionaries. Mathematically, we forced ${∥\alpha^{\left(t\right)}∥}_{2}^{2}=0$ in Eq. (3) and ${∥\alpha^{\left(p\right)}∥}_{2}^{2}=0$ in Eq. (4). In this way, the atoms from *D*^(*p*)^ and *D*^(*t*)^ are incoherent. In addition, ∑i,ji≠j3DiTDjF2 in Eq. (2) is used to promote incoherence between the different dictionaries.^38^ The subscript *F* denotes the Frobenius norm. The energy, i.e., Eq. (2), makes the learning of dictionaries optimal to properly represent the corresponding class via the first and second terms, and forces the remaining class to be weak by the third term. Overall, this function provides a discriminative dictionary.

Our derived objective function Eq. (2) was solved by an alternative optimization process using the DL-COPAR method,^39,40^ which has been widely used in the dictionary learning field. As a result, we obtained the PsP- and TTP-specific dictionaries and a shared dictionary: *D*^(*p*)^, *D*^(*t*)^ and *D*^(*s*)^, respectively.

### 2.E. Construction of discriminative sparse coefficient matrix

**FIG. 3.**
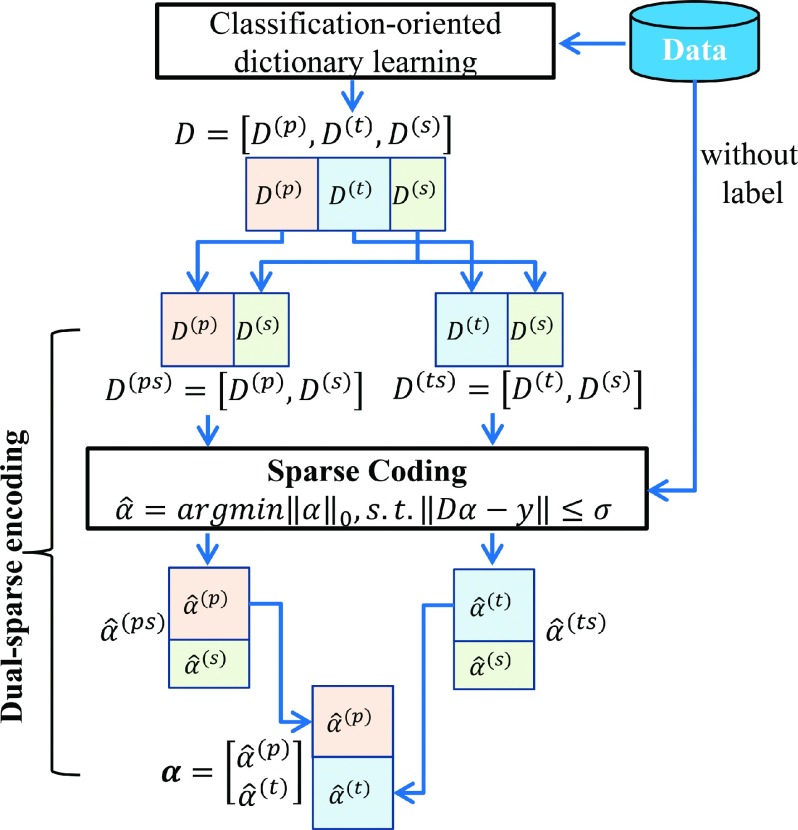
Construction of the discriminative sparse coefficient matrix. *D* = dictionary; *α* = sparse code. PsP, TTP, and the shared patterns are denoted as superscript *p*, *t*, and *s*, respectively.

Intuitively, the PsP data can be sparsely decomposed over the PsP-specialized and the shared dictionaries better than the TTP-specialized and shared dictionaries. To obtain the discriminative sparse coefficients, we proposed a dual-sparse encoding scheme for identical data (Fig. 3)

$${\overset{ˆ}{\alpha }}^{\left(\mathrm{ps}\right)}=\arg\min{∥\alpha^{\left(\mathrm{ps}\right)}∥}_{0},\;\mathrm{s.t.}∥D^{\left(\mathrm{ps}\right)}\alpha^{\left(\mathrm{ps}\right)}-y∥\leq \sigma ,$$ (5)

$${\overset{ˆ}{\alpha }}^{\left(\mathrm{ts}\right)}=\arg\min{∥\alpha^{\left(\mathrm{ts}\right)}∥}_{0},\;\mathrm{s.t.}∥D^{\left(\mathrm{ts}\right)}\alpha^{\left(\mathrm{ts}\right)}-y∥\leq \sigma .$$ (6)

These formulas were approximately solved by greedy pursuit algorithms, such as orthogonal matching pursuit. Here we have $D^{\left(\mathrm{ps}\right)}=\left[D^{\left(p\right)},D^{\left(s\right)}\right]\in R^{m\times \left(K_{p}+K_{s}\right)}$, and its corresponding coefficient ${\overset{ˆ}{\alpha }}^{\left(\mathrm{ps}\right)}=\left[{\overset{ˆ}{\alpha }}^{\left(p\right)};{\overset{ˆ}{\alpha }}^{\left(s\right)}\right]\in R^{\left(K_{p}+K_{s}\right)}$. Similarly, $D^{\left(\mathrm{ts}\right)}=\left[D^{\left(t\right)},D^{\left(s\right)}\right]\in R^{m\times \left(K_{t}+K_{s}\right)}$ and corresponding coefficient ${\overset{ˆ}{\alpha }}^{\left(\mathrm{ts}\right)}=\left[{\overset{ˆ}{\alpha }}^{\left(t\right)},{\overset{ˆ}{\alpha }}^{\left(s\right)}\right]$. For a patch *y*, we sparsely encoded it by *D*^(*ps*)^ and *D*^(*ts*)^, respectively, and obtained the corresponding coefficient ${\overset{ˆ}{\alpha }}^{\left(\mathrm{ps}\right)}$ and ${\overset{ˆ}{\alpha }}^{\left(\mathrm{ts}\right)}$. ${\overset{ˆ}{\alpha }}^{\left(p\right)}$ is the sparse coefficient from the PsP-specific dictionary *D*^(*p*)^, and ${\overset{ˆ}{\alpha }}^{\left(t\right)}$ is the sparse coefficient from the TTP-specific dictionary *D*^(*t*)^. For PsP data, the residual reconstructed by *D*^(*ps*)^ with representation coefficients ${\overset{ˆ}{\alpha }}^{\left(\mathrm{ps}\right)}$ is less than that reconstructed by the *D*^(*ts*)^ with representation coefficients ${\overset{ˆ}{\alpha }}^{\left(\mathrm{ts}\right)}$.

Then, we constructed a new discriminative sparse matrix using the sparse coefficient corresponding to the specific dictionaries and discarding any coefficients regarding the shared dictionary, as follows:

$$\alpha =\left[\begin{array}{c} {\overset{ˆ}{\alpha }}^{\left(p\right)}\\ {\overset{ˆ}{\alpha }}^{\left(t\right)} \end{array}\right],$$ (7)

where $\alpha \in R^{2K}$. *α* has a discriminative characterization. The distributions of ${\overset{ˆ}{\alpha }}^{\left(p\right)}$ were similar in the PsP cases, as seen the distributions of ${\overset{ˆ}{\alpha }}^{\left(t\right)}$ in TTP cases. The new discriminative coefficient matrix was denoted as $A=\left[\alpha_{1},\alpha_{2},\ldots ,\alpha_{N}\right]\in R^{2K\times N}$. This procedure does not require class label information for the data; thus it is unsupervised.

### 2.F. Feature extraction and selection

**FIG. 4.**
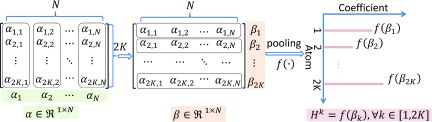
Pooled histogram features from the discriminative coefficient matrix. $f\left(\cdot \right)$ = pooling technique; *α* = sparse code.

Sparse coefficients indicate the contribution of different atoms in the representation of the data, and thus, the sparse coefficients served as the original features of each set of data. First, the discriminative coefficient matrix *A* can also be denoted as $A^{\prime }=\left[\beta_{1};\beta_{2};\cdots ;\beta_{2K}\right]\in R^{2K\times N}$ and $\beta \in R^{1\times N}$, as shown in Fig. 4. *β* describes the different significances of the individual atoms in the construction of overall patches. For example, one element of vector *β* is zero, which means that this atom contributes nothing to the representation of the corresponding patch. Thus, we defined the histogram *H* as a global feature based on atoms.

Formally, each bin *H^k^* associated to the atom *d^k^* of the histogram is computed according to

$$\forall k\in \left[1,2K\right],H^{k}=f\left(\beta_{k}\right)$$ (8)

with $H={\left(H^{k}\right)}_{k\in \left[1,2K\right]}$. $f\left(\cdot \right)$ denoting the pooling technique, which was taken over columns in a single row. In this study, we utilized the max pooling technique, so *H^k^* can be described as

$$H^{k}=\max\left(\beta_{k}\right).$$ (9)

As a consequence, the spatio-temporal images can be described using a histogram. Each bin of the histogram can serve as a local feature, representing the biggest contribution of a corresponding atom in a patient.

The histograms were fed into the classifier directly as global features in the previous dictionary learning classification approaches. Since the differences between PsP and TTP cases are subtle, we applied the feature scoring system (based on the DX score)^34,35^ to identify the most relevant features, i.e., bins of a histogram, with a high degree of discrimination between PsP and TTP. The DX can be mathematically represented as

$$\text{DX}=\frac{{\left(m_{1}-m_{0}\right)}^{2}}{{d_{1}}^{2}+{d_{0}}^{2}}.$$ (10)

In this formula, *m*_1_ and *d*_1_ are the mean value and standard deviation of a feature in the positive samples, while *m*_0_ and *d*_0_ are the corresponding statistics in the negative samples.

Starting from the individual scores, we sequentially added each feature (with DX score from high to low) to form a feature set and tested its predictive performance by tenfold cross-validation. This process yielded a curve of CV accuracy with some top-ranked features. We then identified the best accuracy and the associated features, which would serve as the optimal feature set for next training and testing. The selected features, i.e., atoms with their maximum coefficients, contributed to the discrimination between PsP and TTP.

### 2.G. Classification using SVM algorithm

We chose the SVM algorithm^41^ for classification. Specifically, a well-established SVM tool LIBSVM (Ref. 36) was selected as the classifier. The radial basis function (RBF) kernel was used in the various kernelized learning algorithm based on multiple trials. For each test group, we conducted a grid search on the RBF parameter *γ* and the trade-off coefficient *C*. In addition, the AUC was calculated to evaluate the accuracy and robustness of each classifier. The AUC curve was determined based on sensitivity and specificity, which were calculated as $\text{sensitivity}=\text{TP}/\left(\text{TP}+\text{FN}\right)$ and $\text{specificity}=\text{TN}/\left(\text{FP}+\text{TN}\right)$, where TP, FP, FN, and TN refer to true positive, false positive, false negative, and true negative, respectively.

## 3. EXPERIMENTS AND RESULTS

**FIG. 5.**
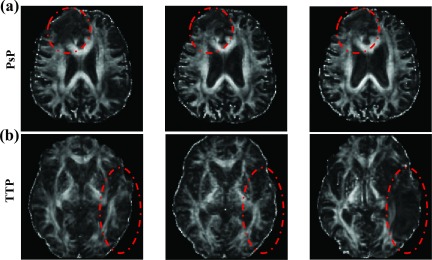
Preprocessing performance of DTI. Registered longitudinal FA images of PsP (a) and TTP (b) at three timepoints (*T*1, *T*2, and *T*3). The dotted line roughly outlines the lesion area.

We collected data from 35 patients with GBM, including 13 cases of PsP and 22 cases of TTP. The longitudinal DTI analyses with three-time points were registered, and the FA images were calculated (Fig. 5). The original image (resolution: 256 × 256) was cropped to 164 × 143 for computational efficiency. Unless otherwise specified, we employed the following empirical parameters for our classification system: patch size *s* = 13, dictionary size of *K_p_* and *K_t_* = 100, *K_s_* = 10% × *K_p_*, *η* = 0.15, and *σ* = *e*^−6^. For all analyses, the number of nonzero coefficients was fixed at 10. We conducted ten rounds of tenfold CV to evaluate the average accuracy of performance. Moreover, we calculated the corresponding AUC to assess the overall performance in terms of robustness of this classification system. *P*-values between different performances were computed using *t*-tests.

### 3.A. Dictionary learning and feature pooling

**FIG. 6.**
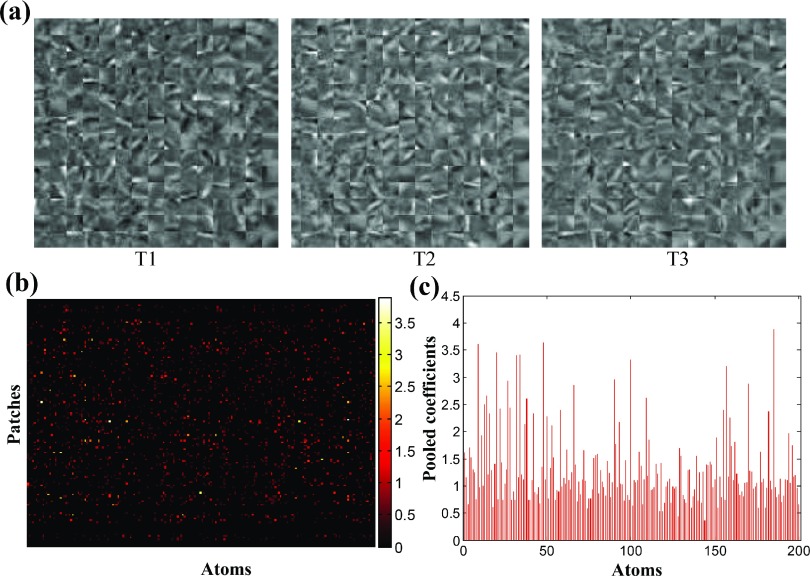
Dictionary learning. (a) Representative PsP-specific dictionary at three-time points. (b) Constructed sparse coefficient matrix. (c) Pooled features from the constructed sparse coefficient matrix shown in (b).

In the dictionary learning stage, we obtained two specific dictionaries of PsP and TTP and one shared dictionary using the classification-oriented dictionary learning method. Figure 6(a) shows a representative PsP-specific dictionary at three-time points. With the twice sparse encoding scheme, the coefficients corresponding to the shared dictionary were excluded, and the remaining data were constructed as a new sparse coefficient matrix, as shown in Fig. 6(b). The sparse coefficient matrices for PsP and TTP were obtained. Since the excluded coefficients from the shared dictionary do not contribute to the discrimination of PsP and TTP, the newly constructed coefficient matrix possesses discriminative attributes. We then applied the max pooling technique to pool the features. The pooled features are shown in Fig. 6(c), i.e., a histogram for all atoms, which can be deemed as a global property of each patient.

### 3.B. Performance of the selected features in discrimination

**FIG. 7.**
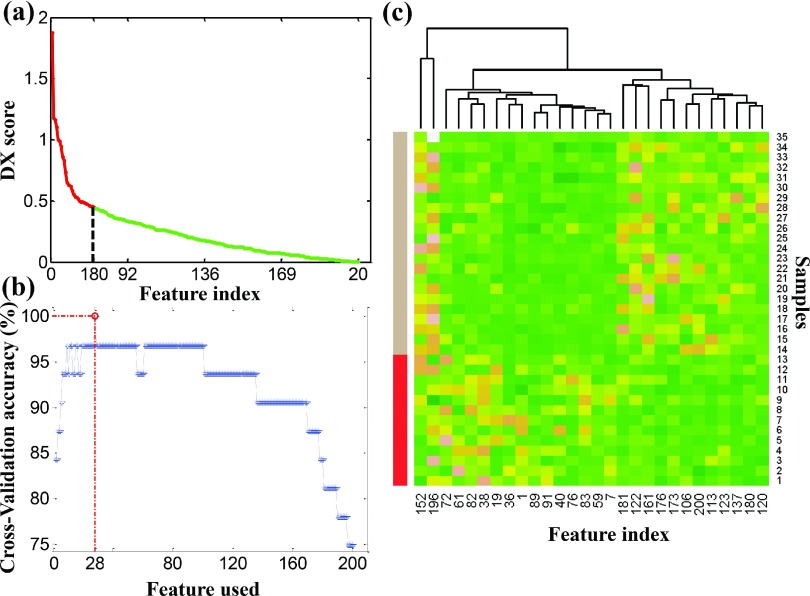
Performance of extracted features in discrimination. (a) DX-scores of the 200 pooled features. (b) Performance after tenfold CV, adding features sequentially. The highest prediction accuracy was achieved using the 28 top-ranked features with tenfold CV and a radial basis function as the kernel function. (c) Hierarchical clustering analysis for the selected 28 features. Samples with labels from 1 to 13 are PsP cases; 14–35 are TTP cases.

The pooled features were sorted by the DX-score [Eq. (10) from high to low, as shown in Fig. 7(a)]. The *x*-axis and *y*-axis represent the feature index and DX-score, respectively. Features with higher scores are better able to distinguish between PsP and TTP according to the definition of DX-score. The ranked features were sequentially added to form a feature set, and the prediction performance was tested by tenfold CV, as shown in Fig. 7(b). Twenty-eight top-ranked features with good predictive accuracy were determined through the analysis of all enrolled patients and served as the optimal feature set for training and testing. This feature selection scheme provided a reasonable performance at a suitable time [*O*(*n*)] while the exhaustive searching took too long to be practical [*O*(2*n*)].

To further illustrate the performance of 28 selected features in discriminating between PsP and TTP, we performed the hierarchical clustering analysis on them using the function heatmap in *R* with default settings. Figure 7(c) shows the hierarchical clustering analysis of the 28 selected features. The sample numbers 1–13 and 14–35 represent the PsP and TTP cases, respectively. The differences between PsP and TTP samples are obvious. Thus, these features can be used to differentiate these two categories.

### 3.C. Performance of the classification system

**FIG. 8.**
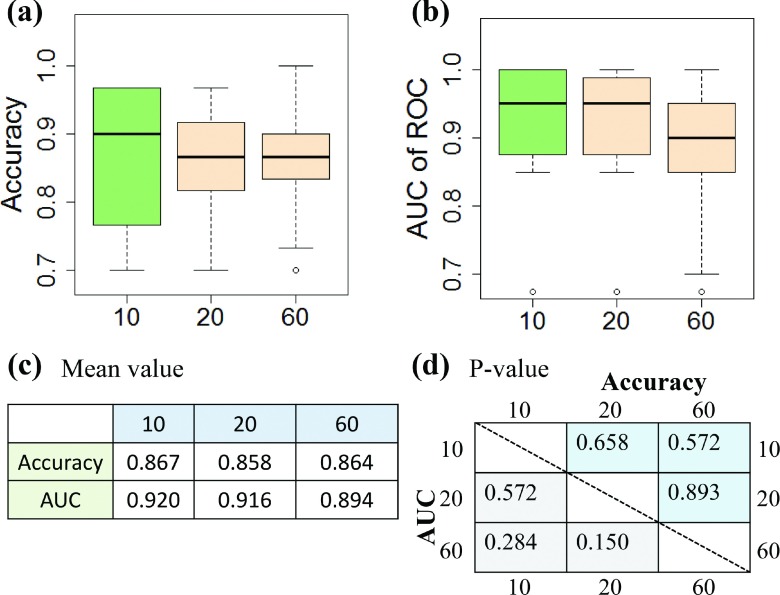
Performance of the classification system. (a) Accuracy; (b) Area under the curve of receiver operating characteristics (ROC); (c) Mean values for accuracy and AUC; (d) *P*-values, calculated with *t*-test, for accuracy and AUC results.

Figure 8 shows the performance of our classification system in terms of accuracies, AUCs, and *p*-values. The average CV accuracies were 0.867, 0.858, and 0.864 with 10, 20, and 60 repetitions, respectively, indicating that our approach has a promising differentiation capability. We also obtained ∼0.9 of AUC values for the performance with various repeated times. In addition, the *p*-values between performances with different repeated times were all greater than 0.15. Therefore, the differences between performances were insignificant, indicating our classification system is stable.

**FIG. 9.**
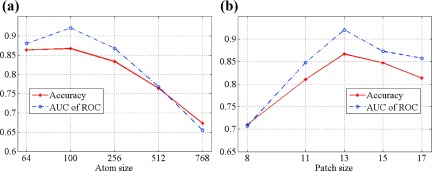
Performance for (a) analyses comparing different dictionary sizes and (b) different patch sizes.

We also analyzed the effects of dictionary and patch sizes on our classification system. Figure 9(a) plots the average accuracy and AUCs with various dictionary sizes (64, 100, 256, 512, and 768). Dictionary sizes less than 500 achieved good results, with accuracies and AUCs greater than 0.8. Accuracies and AUCs for the dictionary size larger than 500 were greater than 0.65 and less than 0.8. The best performance was observed with the dictionary size around 100. Our data indicated that a larger dictionary size did not gain a better performance and was more time-consuming. This observation is consistent with the work of Yang *et al.*^42^ It may be that (1) if the dictionary size is too small, the histograms lose discriminant power and (2) if the dictionary size is too big, the histograms from identical categories are difficult to match. Figure 9(b) shows the effect of patch sizes on the performance of our classification system. The patch size of 13 produced the best performance, with accuracy more than 0.85 and AUC greater than 0.9.

Additionally, we also performed the sensitivity analysis on the weight *η*, patch size *s*, specific dictionary size $K_{p}\left(K_{t}\right)$, and shared dictionary size *K_s_*, as shown in supplementary material Figs. S1–S3.^47^ The relative change in accuracy and AUC was bounded by 10% with the perturbation of these parameters in a range of 20%. Thus, the proposed classification system with our empirical setting was stable.

### 3.D. Comparison with other schemes

#### 3.D.1. Comparisons of the classification system with various data sets

**FIG. 10.**
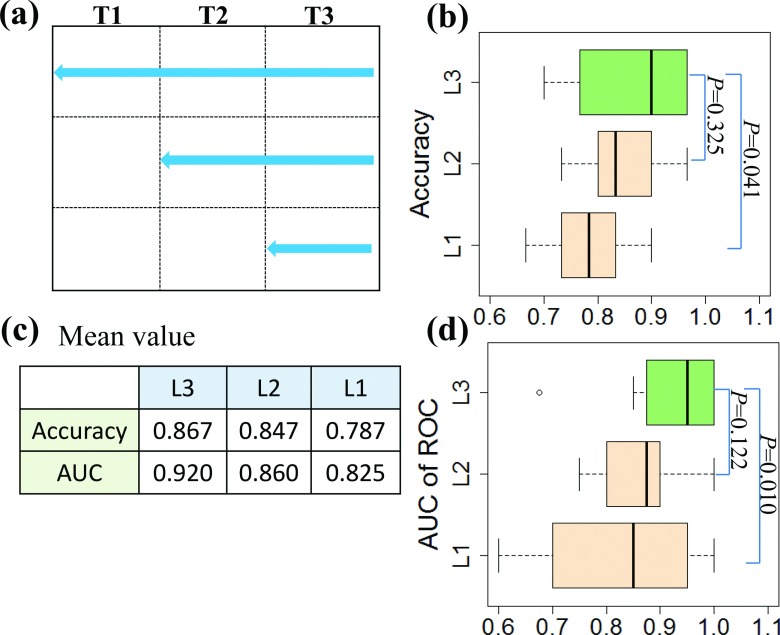
Performance for longitudinal and single data. (a) Depiction of data timepoints; (b) Accuracy; (c) Mean values for accuracy and area under the curve; and (d) AUC of ROC. *P*-values were calculated using *t*-tests.

Our classification system was developed based on clinical follow-up scans from three-time points. We also investigated if using the data from one or two time points could obtain a similar performance. We assumed that *T*3 was the ultimate diagnostic time point, and *T*1 and *T*2 were the previous inspection time points. We defined *L*3 as the data collected from *T*1, *T*2, and *T*3, *L*2 from *T*3 and *T*2, and *L*1 from *T*3 only [Fig. 10(a)]. We tested our approach with the same parameters on the data sets of *L*1, *L*2, and *L*3. The average accuracies and AUCs from ten rounds of tenfold CV are shown in Fig. 10(c). *L*3 had a better performance than other two groups. Although *L*3 performed marginally better than *L*2 in terms of both accuracy and AUC, the difference was insignificant since the *p*-values were greater than 0.05 [Figs. 10(b) and 10(d)]. AUC value for *L*1 was 0.825. However, *L*3 outweighs *L*1 significantly (*p* < 0.05). Our analysis indicates that the longitudinal data boost the classification performance.

#### 3.D.2. Effects of different pooling techniques on performance

In the computer vision field, several pooling techniques have been used for classification based on dictionary learning. To determine if other pooling techniques have more advantages than the max pooling technique we used, we tested the predictive accuracy of our system with other four pooling methods, including square root of mean squared statistics (Sqrt), mean of absolute values (Mean), summary of absolute values (Sum), and the combination of max, Sqrt, Mean, Sum pooling techniques (Comb). Sqrt, Mean, and Sum were defined as

$$\text{Sqrt}:H^{k}=\sqrt{\frac{1}{N}\overset{N}{\underset{i=1}{\sum }}\alpha_{k,i}^{2}},\text{Mean}:H^{k}=\frac{1}{N}\overset{N}{\underset{i=1}{\sum }}\left|\alpha_{i,k}\right|,\text{Sum}:H^{k}=\overset{N}{\underset{i=1}{\sum }}\left|\alpha_{i,k}\right|.$$ (11)

**FIG. 11.**
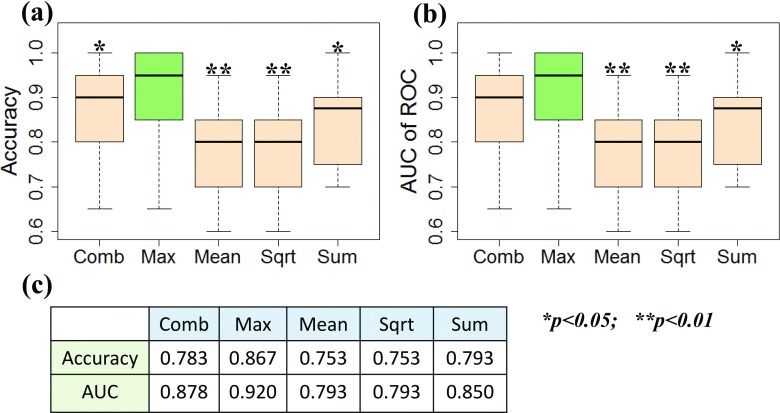
Performance for different pooling techniques. (a) Accuracy; (b) Area under the curve (AUC); and (c) Mean values for accuracy and AUC. *P*-values were calculated using *t*-tests.

The average accuracies and AUCs from ten rounds of tenfold CV using five pooling techniques are shown in Fig. 11. The accuracies ranged from 0.753 to 0.867 [Figs. 11(a) and 11(c)] and AUC values from 0.793 to 0.92 [Figs. 11(b) and 11(c)]. The max pooling technique produced better classification results with accuracy and AUC values 0.867 and 0.92, respectively, probably attributable to its robustness to local spatial variations.

#### 3.D.3. Performance using locally linear embedding (LLE) for feature extraction

Dimensionality reduction is a key step in a classification system. In our system, we applied the feature scoring system^34,35^ for feature selection. Nonlinear dimensionality reduction, such as LLE, has been used by others previously.^43^ Here, we investigated whether LLE could perform well in our proposed classification scheme. We applied LLE to extract 4, 8, and 16 dimensions from hundreds of dimensions. These reduced dimensions hold most of the discriminative information. Table I shows the performance of LLE in our classification system. All of the AUC values from three dimensions were greater than 0.8, but all of the accuracy values were less than 0.8. Comparatively, the feature scoring system performed better than LLE regarding the classification accuracy and AUC.

**TABLE I.** Performance of LLE in the proposed classification system. AUC = area under the curve; SD = standard deviation.

	Accuracy		AUC	
Dimension	Mean	SD	Mean	SD
4	0.760	0.091	0.828	0.321
8	0.770	0.199	0.875	0.276
16	0.753	0.220	0.805	0.317

#### 3.D.4. Performance using FA values as immediate features

Clinically, the longitudinal FA values can convey the diagnostic information for PsP and TTP. In this study, we investigated the classification ability of FA values as immediate features. First, the average FA value, i.e., $\bar{y}=\sum_{i}^{m}a_{i}/m$, of each image patch was calculated as a feature, where *y* is a 3D patch, *m* is the length of column-vector, and *a_i_* is a FA value associated to the pixel *i*. Hence, we obtained a feature vector composed by the average FA values of the image patches for each case. Then, we selected the most discriminative features using the feature scoring system. Table II shows the ten rounds of tenfold cross-validation results. The accuracy and AUC values were quite low, indicating that FA values as immediate features are not suitable for the classification of PsP and TTP. Therefore, the dictionary learning method is a good choice for making the full use of the DTI in differentiating between PsP and TTP.

**TABLE II.** Performance using FA values as immediate features. AUC = area under the curve; SD = standard deviation.

	Mean	SD	Maximum	Minimum
Accuracy	0.603	0.210	1	0
AUC	0.445	0.417	1	0

#### 3.D.5. Performance using morphological features from MRI

Morphological changes in tumor regions on follow-up MRI have been used as indicators for the development of PsP and TTP. Therefore, we explored the classification performance of morphological features from the longitudinal MRI data for PsP and TTP. We collected data from 17 patients with GBM (5 PsP, 12 TTP) who had longitudinal contrast-enhanced T1 Flair MRI scans. First, we segmented the enhancing and necrotic areas using *s* semiautomatic segmentation scheme, as shown in supplementary material Fig. S1.^47^ Then, we extracted 225 morphological features from segmented tumor regions (supplementary material Table S1),^47^ such as the major/minor axis length of the enhanced region and the thickness of enhancing margin. The details of the semiautomatic segmentation and feature extraction are shown in the supplementary material.^47^ The classification accuracy was 0.615 and AUC was 0.72, as shown in Table III. Our analysis indicates that the morphological features of tumor regions have limited discriminative capability for PsP and TTP. This is consistent with the conclusion from a previous study.^13^

**TABLE III.** Performance of morphologic features from longitudinal MRI. AUC = area under the curve; SD = standard deviation.

	Mean	SD	Maximum	Minimum
Accuracy	0.615	0.245	1	0
AUC	0.720	0.451	1	0

## 4. DISCUSSION

Differentiation of PsP and TTP in GBM patients is still challenging for physicians since PsP can mimic tumor progression at the tumor site or resection margins, and its appearance on MRI is comparable to that of TTP. Genetic biomarkers and imaging features have been explored to distinguish PsP from TTP. However, the previous work has limitations, and the results to date are equivocal. So far, there are no biomarkers or computer-aided diagnosis systems utilized clinically for this purpose. Thus, there is an urgent need for the development of new approaches to improve the accuracy and efficiency of diagnosis. In this study, we developed an objective classification system to distinguish between PsP and TTP based on spatio-temporal dictionary learning with longitudinal DTI. DTI has a promising potential in differentiating the two phenotypes, and longitudinal DTI scans used in the proposed system are analogous to the clinical strategy of follow-up surveillance. To best of our knowledge, it is the first time that a dictionary learning scheme has been used to differentiate between PsP and TTP. Hence, the extracted features used in our classification system are independent on the segmentation of ROI. In addition, we constructed a novel discriminative sparse matrix to exclude features that are shared between PsP and TTP. The ultimate selected features based on the discriminative sparse matrix captured subtle differences between PsP and TTP. Our classification system was confirmed by tenfold CV and AUC analysis.

The main contribution of this study is to introduce the use of a dictionary learning scheme for the diagnosis of PsP versus TTP in patients with GBM. The objective classification system based on dictionary learning can directly extract the features from the sparse coefficient matrix by pooling techniques, and hence, it can avoid the segmentation of ROI. In the traditional classification system, the ROI segmentation is a fundamental and critical step, since the classification performance is dependent on the characteristics of ROI, which is subject to the segmentation quality. The lesion segmentation in MRI/DTI is an open area in the research community, in particular for the segmentation of GBM recurrence area, due to the low contrast and multiple lesion areas. The current method of manual identification of ROI for the differentiation of PsP and TTP is labor-intensive, probably unreliable, and often infeasible for large-scale studies. Therefore, a dictionary learning scheme is a rational solution for the differentiation of PsP and TTP.

Another significant strategy of this work was our construction of a discriminative sparse matrix for the feature extraction. We used a classification-oriented dictionary learning method to obtain three types of specific dictionaries, i.e., *D*^(*p*)^, *D*^(*t*)^ and *D*^(*s*)^, for PsP, TTP, and shared patterns, respectively, on the training samples with labels. To eliminate labels at the sparse coding stage, we proposed a dual sparse encoding scheme for training and testing of samples. Specifically, we sparsely encoded the samples by the combination of PsP-specific and shared dictionaries (i.e., $\left[D^{\left(p\right)},D^{\left(s\right)}\right]$) and the combination of TTP-specific and shared dictionaries (i.e., $\left[D^{\left(t\right)},D^{\left(s\right)}\right]$), respectively, and obtained the corresponding coefficient matrices $\left[\alpha^{\left(p\right)},\alpha^{\left(s\right)}\right]$ and $\left[\alpha^{\left(t\right)},\alpha^{\left(s\right)}\right]$. For the individual samples without labels, we obtained two coefficient matrices based on the trained dictionaries. We then extracted the coefficients corresponding to the specific dictionaries and constructed a new sparse matrix, i.e., $\left[\alpha^{\left(p\right)};\alpha^{\left(t\right)}\right]$. This constructed sparse matrix contains more discriminative information attributed to exclude the shared features. The coefficient matrix including the shared features, i.e., $\left[\alpha^{\left(p\right)};\alpha^{\left(s\right)};\alpha^{\left(t\right)};\alpha^{\left(s\right)}\right]$, produced the average accuracy and AUC of 0.863 and 0.905 by ten rounds of tenfold cross-validation. The AUC value was 1.5% higher, when constructing the sparse matrix excluding the shared features.

Dimensionality reduction of pooled features is necessary for obtaining good classification results. The pooled features from the sparse matrix have hundreds of dimensions, which are related to the size of a dictionary. Some studies based on dictionary learning, however, have directly fed the pooled histogram features into classifiers without the feature dimension reduction and obtained reasonable performance.^42^ Our classification system gained an average accuracy of 0.867 with feature selection but 0.736 without feature selection in terms of tenfold CV, indicating that the classification system with a dimensionality reduction performed better. In addition, the nonlinear dimensionality reduction has attracted extensive attentions, especially LLE. LLE is an unsupervised learning algorithm that computes the low-dimensional analyses of high-dimensional data but preserves neighborhood embedding. We compared the performance of LLE with our feature scoring system and found that all of AUC values were greater than 0.8, while accuracies were lower than 0.8 in LLE. Obviously, our feature scoring method is better for feature selection than LLE regarding the classification accuracy and AUC. These analyses demonstrate that the feature scoring approach is optimal for our classification system in the differentiation of PsP and TTP.

The discriminating capability of the features identified by the feature scoring system was investigated in this study. Histogram is the global characteristic and represents the different significances for all of the atoms in an individual case. The selected features by our scoring system are the discriminative atoms with their maximum weights. As shown in Fig. 7(c), these discriminative atoms essentially contribute to the differentiation of the two phenotypes. Specifically, the intensity (i.e., FA value) differences between PsP and TTP can be reconstructed by the identified atoms with maximum weights. This mechanism of our classification system is similar to the clinical imaging analysis of FA in distinguishing PsP and TT.^20,21^

**FIG. 12.**
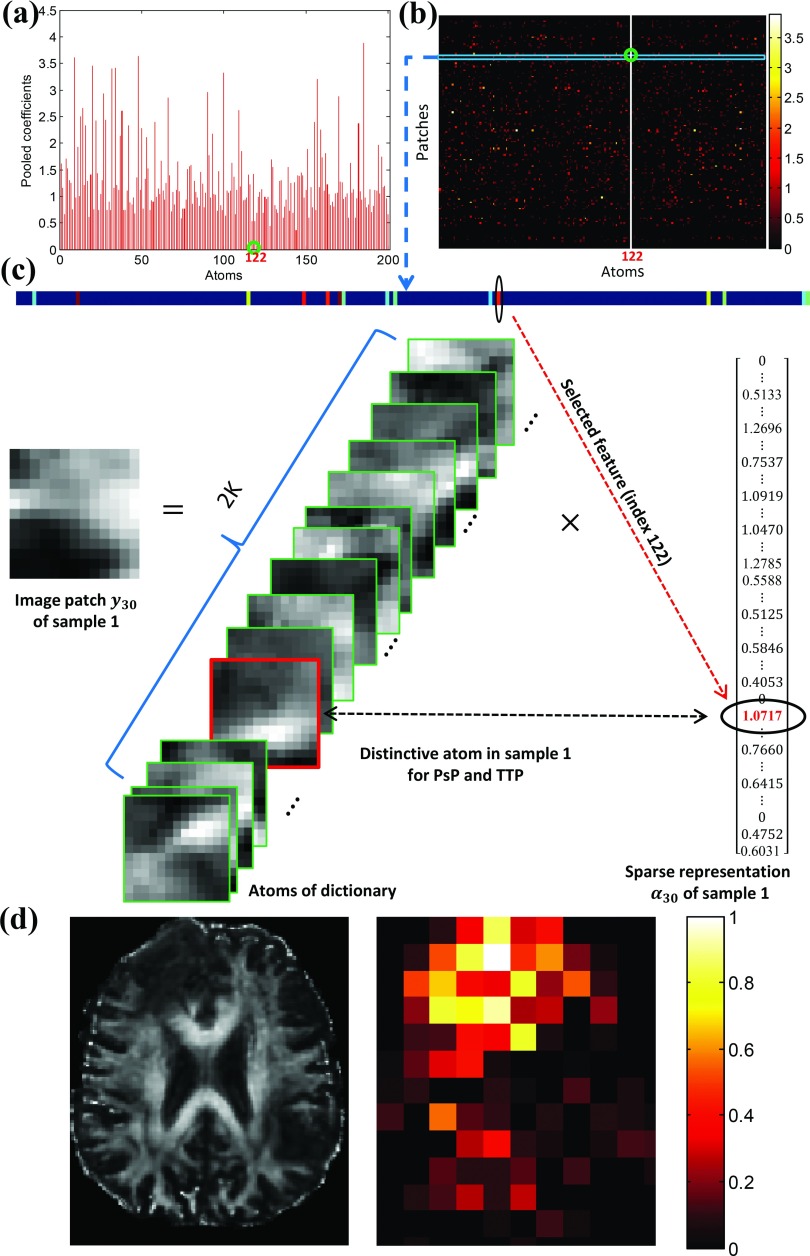
Physical meaning of features. (a) A representative feature, i.e., the maximum sparse coefficient of the 122nd atom in the sample number 1, selected by the feature scoring system on the histogram (pooled features); (b) the maximum sparse coefficient of the 122nd atom corresponds to the 30th patch; (c) among 15 nonzero coefficients, the 122nd coefficient is the selected unique feature and contributes to the intensity difference between PsP and TTP for the 30th image patch; (d) a representative image (left) and the distribution of discriminative image patches selected by ten rounds of tenfold CV (right).

Figure 12 gives a further explanation of the physical meaning of the identified features. The maximum sparse coefficient of the 122nd atom [Fig. 12(b)] is the selected feature and corresponds to the 30th patch of the 1st image [Fig. 12(a)]. The 30th patch can be a linear combination of the atoms. There are 15 nonzero sparse coefficients corresponding to the 30th patch, as shown in Fig. 12(c). Among these nonzero coefficients, the maximum sparse coefficient of the 122nd element is the unique selected feature. Thus, the 30th image patch of the 1st sample is the discriminative patch between PsP and TTP. In this discriminative patch, the intensity created by the 122nd atom and its maximum coefficient reflects the difference in FA levels in these two entities. Furthermore, most of the discriminative patches identified by the selected features are located in or near the pathological area, as shown in Fig. 12(d). This phenomenon is rational,^44,45^ as the difference intensity, which was contributed by the identified features, reflects the FA difference in pathological areas between the two groups on DTI.

We also checked the performance of our classification system with the spatial pyramid matching (SPM) technique,^46^ which has been widely used in the dictionary learning classification systems.^30,42^ We partitioned the image into three levels of 0, 1, and 2, with 1, 4, and 16 segments. Thus, we had a total of 21 pooled feature vectors which were reshaped into one feature vector for the final representation. The average accuracy of SPM was 0.776, and the AUC was 0.853 after ten rounds of tenfold CV. Although the performance was good, SPM did not advance the performance of our classification scheme for PsP and TTP.

## 5. CONCLUSION

To conclude, we developed an objective classification approach without segmentation to distinguish PsP and TTP. The longitudinal DTI, as a promising imaging approach for characterizing microstructural changes or differences in neuropathology and treatment, was utilized in this method. The spatio-temporal and classification-oriented dictionary learning framework was applied to capture the subtle discriminative characteristics between PsP and TTP. The feature extraction is independent of segmentation of ROI. The results demonstrated that our proposed system can achieve the reliable and accurate classification of PsP and TTP. Therefore, this system will be a useful tool in the clinical diagnosis of PsP and TTP.
